# Molecular engineering on a MoS_2_ interlayer for high-capacity and rapid-charging aqueous ion batteries†

**DOI:** 10.1039/d3na00068k

**Published:** 2023-04-04

**Authors:** Xuefei Han, Jing Yang, Yong-Wei Zhang, Zhi Gen Yu

**Affiliations:** a Institute of High Performance Computing (IHPC), Agency for Science, Technology and Research (A*STAR) 1 Fusionopolis Way, #16-16 Connexis Singapore 138632 Republic of Singapore zhangyw@ihpc.a-star.edu.sg yuzg@ihpc.a-star.edu.sg; b Department of Materials Science and Engineering, National University of Singapore Singapore 117575 Singapore; c AVIC Xi'an Flight Automatic Control Research Institute 710065 China

## Abstract

Rechargeable aqueous ion batteries (AIBs) play essential roles in the increasing demand for high-performance energy storage systems, and yet they are hampered by the lack of suitable cathode materials because of the sluggish intercalation kinetics. In this work, we develop an effective and feasible strategy to enhance the performance of AIBs by broadening the interlayer spacing by using intercalated CO_2_ molecules to promote the intercalation kinetics by using first principles simulations. Compared with pristine MoS_2_, the intercalation of CO_2_ molecules with a 3/4 ML coverage significantly increases the interlayer spacing to 9.383 Å from 6.369 Å and the diffusivity is boosted by 12 orders of magnitude for Zn ions, 13 orders for Mg ions and one order for Li ions. Moreover, the concentrations of intercalating Zn, Mg and Li ions are enhanced by 7, 1 and 5 orders of magnitude, respectively. The significantly increased diffusivity and intercalation concentration of metal ions signify that intercalating CO_2_ bilayer MoS_2_ is a promising cathode material to realize metal ion batteries with a rapid charging capability and high storage capacity. The strategy developed in this work can be generally applied to increase the metal ion storage capacity in transition metal dichalcogenide (TMD)- and other layered material-based cathodes and make them promising for next-generation rapidly rechargeable batteries.

## Abstract 2

Intercalated CO_2_ molecules can expand the interlayer spacing of layered materials, resulting in high-capacity and rapid-charging aqueous ion batteries.

## Graphical abstract

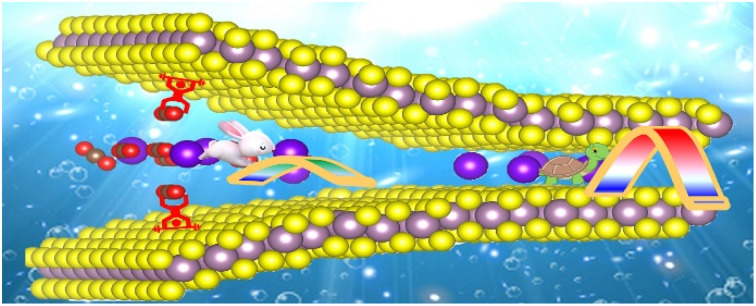

## Introduction

Renewable and clean energy generation is a promising solution to counter the carbon dioxide emission mainly originating from burning fossil fuels. Several renewable energy harvesting technologies have been well developed.^1–5^ The rapid development and high demands of renewable and clean energy sources, portable electronic devices, and electric vehicles have triggered great ambition for low cost, large-scale, and high energy density battery systems for energy storage. Due to the high cost and safety hazards and the scarce source of metal Li, it is desirable to find alternative energy storage systems to replace Li-ion batteries (LIBs). In this regard, aqueous multivalent metal ion batteries (AMMIBs) are attracting tremendous attention and are considered promising substitutes for LIBS.^6–13^ Due to high safety, low cost, eco-friendliness, and high ionic conductivity (1000 times higher than organic electrolytes), rechargeable AMMIBs are promising batteries for grid-scale electrochemical energy storage. Among rechargeable AMMIBs, aqueous zinc-ion batteries (ZIBs)^14–17^ and aqueous magnesium-ion batteries (MIBs)^18–20^ have attracted remarkable attention worldwide because they exhibit a high volumetric energy density of 5851 mA h mL^−1^ for ZIBs^21,22^ and 3833 mA h mL^−1^ for MIBs.^23–25^ However, the main challenge lies in developing suitable cathode materials for AMMIBs.

Several materials have been developed as promising cathode materials for AMMIBs. Vanadium oxides with a tunnel structure as cathode materials show high Zn-ion storage properties,^26–31^ but the dissolution of vanadium in water-based electrolytes remains a significant challenge.^32^ A composite of manganese dioxide and carbon molecular sieves (δ-MnO_2_@CMS) with a core–shell structure and Chevrel phase Mo_6_S_8_ were reported as cathode materials for MIBs.^33–35^ The practicality of MIBs is hampered by the absence of suitable high-performance cathode materials with rapid Mg ion diffusion.^36^ Transition metal dichalcogenides (TMDs) have attracted extensive attention due to their potential applications in cathode materials for rechargeable AMMIBs. The layer structure of TMDs and their weak interlayer interaction *via* van der Waals (vdW) force are appealing properties for multivalent metal ion diffusion and intercalation,^37–41^ especially for large metal ion-based AMMIBs. Among TMDs, MoS_2_ is considered one of the most promising cathode materials for AMMIBs,^42,43^ which suggests the feasibility of employing MoS_2_ as a functional AMMIB cathode material. This scarcity in the report suggests the heightened challenges in observing reversible metal ion storage in pristine MoS_2_. The high intercalation energy barrier of metal ions leads to the low specific capacities recorded for the MoS_2_ systems (for Zn 1–40 mA h g^−1^).^44–46^ For example, it was found that Zn^2+^ diffusion across the MoS_2_ framework was further hampered by its low electrochemical activity and low conductivity.^45^ Hence, it is essential to explore effective modification methods to “activate” MoS_2_ towards reversible metal ion storage for leveraging the advantages of MoS_2_. Phase engineering was a highly appealing strategy for modulating the chemical and electrical properties. 1T-MoS_2_, with a tetragonal symmetry, in which each Mo atom has an octahedral coordination with S atoms, has metallic conductivity, resulting in a lower metal ion diffusion barrier than in 2H-MoS_2_. However, 1T MoS_2_ is a metastable phase, and producing 1T MoS_2_ on a large scale remains a big challenge. An alternative strategy is to tune the intercalation energy by changing the interlayer spacing. It was demonstrated that intercalation oxygen could increase their interlay spacing (9.5 Å) and tune hydrophilicity, resulting in boosting the Zn ion diffusion kinetics by 3 orders of magnitude in MoS_2_.^44^ Sandwiched structures consisting of monolayer MoS_2_ and carbon (MoS_2_ : C)^47^ and MoS_2_/graphene^48^ were reported to realize high-performance sodium ion and Zn ion batteries thanks to the expanded interlayer spacing (11.6 Å). Although many efforts have been made to study layered MoS_2_, the high capacity and long life-cycle times of MoS_2_-based cathodes have not yet been coexisting for practical utilization. More work needs to be conducted to expand the interlayer spacing further and enhance the hydrophilicity of MoS_2_ to realize high reversible capacity and superb durability, which remains a big challenge.

In this study, we demonstrate a feasible and effective strategy to reduce metal ion diffusion barrier by using intercalation of CO_2_ molecules to expand the MoS_2_ interlayer spacing through density functional theory (DFT) simulations. Our comprehensive DFT results reveal that the intercalated MoS_2_ by CO_2_ is a promising cathode material for realizing rapidly chargeable metal ion batteries.

## Results and discussion

*AA*′ stacking bilayer MoS_2_ was chosen to investigate the metal ion diffusion in this study since it has the lowest relative formation energy among five possible stacking configurations.^49^ The optimized unit cell of *AA*′ stacking bilayer MoS_2_ is shown in Fig. S1a.† The optimized lattice constants are *a* = *b* = 3.204 Å and the layer spacing is 6.37 Å, which is quite close to the reported value of 6.21 Å.^49^ The stacking formation *E*_S_ of bilayer MoS_2_ was calculated based on the definition of the total energy difference per atom between the bilayer and two individual monolayers 
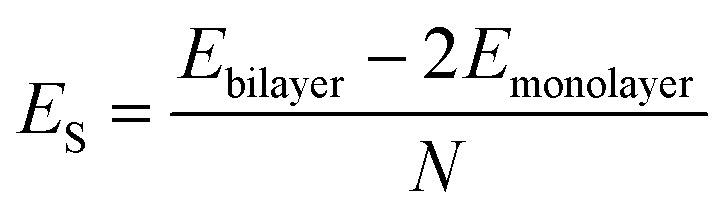
, where *N* is the total number of atoms in bilayer MoS_2_ unit cell (4 S and 2 Mo atoms). The calculated *AA*′ stacking formation energy is −34.33 meV per atom, well agreeing with the reported value of −25.13 meV.^49^ The tiny difference may originate from the van der Waals (vdW) correction methods.^50^ The optimized unit cell was expanded to build 5 × 5 × 1 supercells containing 50 Mo and 100 S atoms shown in Fig. S1b,† and rectangle supercells (*a* = 22.201 Å and *b* = 19.224 Å) with 96 Mo and 192 S atoms shown in Fig. S1b.†

**Fig. 1:**
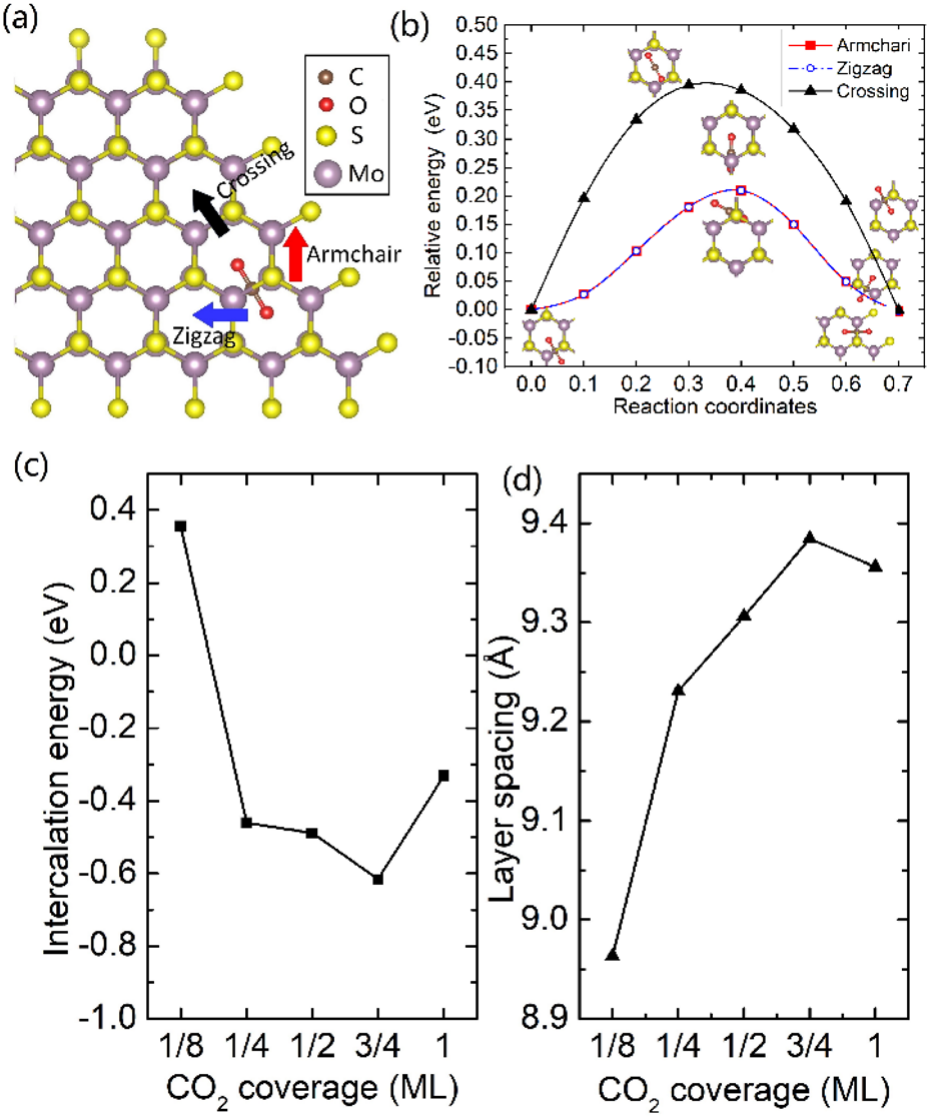
(a) Three possible diffusion pathways of the intercalation of CO_2_ molecule. (b) The diffusion barrier of CO_2_ in bilayer MoS_2_. The CO_2_ molecule coverage-dependent intercalation energies (c) and layer spacing (d).

It was well explored that the interlayer spacing and coupling have a strong effect on the intercalation energy of metal ions.^44,47,48^ In this study, the intercalating CO_2_ molecules were used to expand the interlayer spacing of bilayer MoS_2_. First, the possible embedding configurations of the intercalating CO_2_ molecules were investigated, and the optimized configurations and the calculated relative energies are shown in Fig. S2.† In which, the CO_2_ molecule has two possible embedding sites, and the computed relative energies reveal that CO_2_ prefers to stay at the bridge site rather than the hollow site. The intercalation energy of an intercalating CO_2_ molecule *E*_In_ was calculated based on *E*_In_ = *E*_(MoS_2_+CO_2_)_ − (*E*_MoS_2__ + *E*_CO_2__), where, *E*_(MoS_2_+CO_2_)_, *E*_MoS_2__ and *E*_CO_2__ are the total energies of bilayer MoS_2_ with one embedding CO_2_ molecule, pristine bilayer MoS_2_ and an isolated CO_2_ molecule. For an isolated CO_2_ molecule energy calculation, we put one CO_2_ molecule in a cube model (*a* = *b* = *c* = 15 Å), and the calculated ground state energy is considered the energy of an isolated CO_2_ molecule. The calculated intercalation energy of one embedding CO_2_ molecule is 2.98 eV at the bridge site. With one intercalating CO_2_ molecule, the interlayer spacing slightly increases to 7.61 Å from 6.37 Å (pristine bilayer MoS_2_). The relatively high intercalation energy of the CO_2_ molecule may originate from the strong interlayer coupling. Therefore, more CO_2_ molecules may need to be intercalated to expand the interlayer spacing and reduce the interlayer coupling further. Meanwhile, we also investigate the intercalating CO_2_ molecule diffusion in bilayer MoS_2_. According to the symmetry of the 2H MoS_2_ structure, the intercalating CO_2_ has three possible diffusion pathways: armchair, zigzag and crossing, as shown in Fig. 1a. The calculated diffusion barriers are 0.209, 0.209 and 0.394 eV as shown in Fig. 1b. The computed results reveal that the CO_2_ diffusion barriers along the zigzag and armchair directions are identical, revealing an isotropic diffusivity of the intercalating CO_2_ in the bilayer MoS_2_. Also, the direct crossing diffusion is more difficult due to its higher diffusion barrier than zigzag and armchair directions originating from the high relative energy at the hollow site. The calculated diffusion barriers of 0.209 and 0.394 eV also show that the intercalation CO_2_ would be kinetically stable in the bilayer MoS_2_ since the room temperature is about 0.0256 eV. Temperature and pressure contributions are not included in the results obtained from DFT simulations, which only are valid at *T* = 0 K and *P* = 0 atm. The results from DFT simulations can be used as an input to thermodynamics considerations to describe a situation of finite temperature and pressure. To investigate the thermodynamic stability of the CO_2_ intercalated MoS_2_, we performed DFT simulations considering appropriate thermodynamic functions, and the CO_2_ intercalated stacking Gibbs free energy as a function of the temperature and pressure can be calculated

1 Δ*G*(*T*, *P*) = *E*_S_ + Δ*E*_ZPE_ − *T*Δ*S*_vib_ + *PV*

where *E*_S_ is the normalized CO_2_ intercalated stacking energy directly from DFT simulations, defined as

2 *E*_S_ = (*E*_(MoS_2_+*n*CO_2_)_ − (*E*_(MoS_2_)_ + *nE*_CO_2__))/*n*

where *E*_(MoS_2_+*n*CO_2_)_, *E*_(MoS_2_)_ and *E*_CO_2__ are the energies of monolayer or bilayer MoS_2_ with *n*CO_2_ molecules, a clean monolayer, or bilayer MoS_2_ and the CO_2_ molecule, respectively. *E*_ZPE_ and *S*_vib_ are the zero point energy and the entropy contributed by vibration frequency *ω*_*i*_ and *T* = 298 K. *E*_ZPE_ can be calculated using 
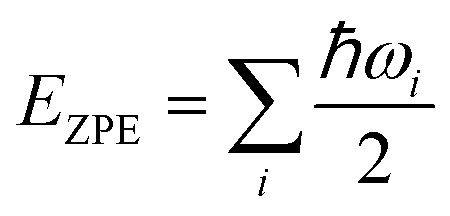
, and *S*_vib_ can be calculated using 
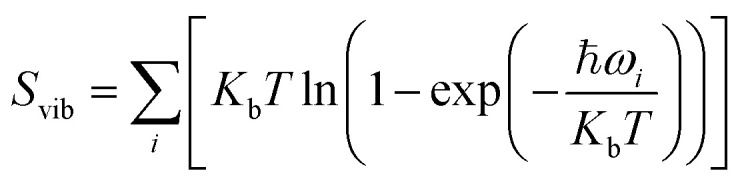
, where ℏ is the reduced Planck's constant and *ω*_*i*_ is the *i*-th vibrational frequency of the diffusing ion. As a benchmark, we computed the entropy CO_2_ molecule and the calculated entropy is 214.419 J (mol^−1^ K^−1^), which is comparable with the standard molar entropy of 213.79 J (mol^−1^ K^−1^). In this study, we proposed two pathways to intercalate CO_2_ into MoS_2_, directly intercalating CO_2_ molecules into one bilayer MoS_2_ (P1) or two monolayers (P2) one by one as shown in Fig. S6a.† Under the condition of *P* = 0 atm, we calculated the CO_2_ intercalated stacking Gibbs free energies, and the results are shown in Fig. S6b.† In comparison, using two monolayers (P2) may easily realize the CO_2_ intercalated MoS_2_. As shown in Fig. S6b,† the stacking Gibbs free energy of one CO_2_ intercalated MoS_2_ is 3.48 eV using the P1 pathway and dramatically decreases to −3.50 eV. When the CO_2_ coverage increases to 3/4 and 1 ML, the stacking Gibbs free energies of the CO_2_ intercalated MoS_2_ are 0.36 and 0.26 eV using the P1 pathway, much higher than those of −0.16 eV and −0.13 eV using the P2 pathway, respectively. Based on the definition of the stacking Gibbs free energy, the negative value denotes that the final product of the CO_2_ intercalated MoS_2_ is thermodynamically stable, and the reaction is exothermic. It should be noted that the calculated stacking Gibbs free energies of the CO_2_ intercalated MoS_2_ are based on *P* = 0 atm. Based on eqn (1), we may estimate the pressure needed to realize 3/4 ML CO_2_ intercalation when the Gibbs free energy is zero using the P1 process. The estimated pressure is 107 atm (0.0108 GPa) to stabilize CO_2_ molecule (3/4 ML) intercalating in bilayer MoS_2_. Furthermore, intercalating one CO_2_ molecule may require higher pressure (*P* = 961 atm). Based on the results shown in Fig. S6b,† it can be seen that the intercalated structure appears unstable at a low coverage and becomes more stable at a high coverage, which reveals a substantial kinetic barrier in realizing the CO_2_ molecules intercalated structure. Not surprisingly, the calculated CO_2_ diffusion barriers of 0.209 and 0.394 eV shown in Fig. 1b can be considered the kinetic barrier for realizing a stable structure at a high coverage. The diffusion barriers also reveal that the intercalating CO_2_ would be kinetically stable in the bilayer MoS_2_ since the diffusion barriers are much higher than 0.0256 eV (room temperature). Also, we performed *ab initio* molecular dynamics (AIMD) for one CO_2_ molecule intercalated MoS_2_ and bilayer MoS_2_ with a CO_2_ coverage of 1 ML at 300 K within 1000 fs. Fig. S7† shows intercalated CO_2_ molecules stabilized in the bilayer MoS_2_, maintaining the 2H structural symmetry. The optimized models obtained from AIMD simulations also confirm the thermodynamical stability. Therefore, the CO_2_ intercalated MoS_2_ is thermodynamically stable and feasible. The diffusion barrier also indicates that intercalating CO_2_ molecules can easily form a uniform distribution in bilayer MoS_2_. The relative energy results also reveal that intercalating CO_2_ molecules prefer to separate rather than cluster in the bilayer MoS_2._

To expand the interlayer spacing and reduce the interlayer coupling further, more CO_2_ molecules were intercalated into the bilayer MoS_2._ The CO_2_ coverage-dependent intercalation energy *E*_f_ was computed based on the definition of *E*_f_ = *E*_(MoS_2_+*n*CO_2_)_ − *E*_(MoS_2_+(*n*−1)CO_2_)_ − *E*_CO_2__, where *E*_(MoS_2_+*n*CO_2_)_, *E*_(MoS_2_+(*n*−1)CO_2_)_ and *E*_CO_2__ are the energies of nCO_2_ intercalating into MoS_2_, (*n* − 1)CO_2_ intercalating into MoS_2_ and an isolated CO_2_ molecule. In this study, we only consider the CO_2_ coverage of 1/8 ML, 1/4 ML, 1/2 ML, 3/4 ML and 1 ML (24 CO_2_ molecules) using big rectangle supercells composed of 96 Mo and 192 S atoms, and the optimized models are shown in Fig. S5.† The calculated CO_2_ molecule coverage-dependent intercalation energies and the corresponding layer spacings are shown in Fig. 1c and d. The simulation results show that the intercalation energy decreases with the increase in coverage, and the 3/4 ML coverage of the CO_2_ molecules results in the lowest intercalation energy of −0.615 eV, and the intercalation energy is increased when the CO_2_ coverage further increases. Not surprisingly, the interlayer spacing increases with the increase in coverage, resulting in the largest layer spacing of 9.384 Å among five considered coverages. A further increase in coverage (1 ML) makes the interlayer spacing decrease when the CO_2_ coverage is higher than 3/4 ML. Based on the results shown in Fig. S5,† the CO_2_ intercalated MoS_2_ keeps the structural stability with increased coverage even at the coverage of 1 ML. The calculated *AA*′ stacking formation energy of bilayer MoS_2_ with a CO_2_ molecule coverage of 3/4 ML increases to −28.01 meV per atom, which is higher than that in pristine MoS_2_ of −34.33 meV per atom. A comparison of these results reveals that the intercalation of CO_2_ weakens the layer coupling. The calculated interlay spacing of the bilayer MoS_2_ with 3/4 ML coverage is 9.384 Å, slightly higher than that with a 1 ML coverage of 9.356 Å. It should be noted that the interlay spacing is the average distance between two Mo atoms in the different layers. Based on the results shown in Fig. 1d, we may consider that the expanded interlayer spacing is converged when the CO_2_ coverage is more than 3/4 ML. Therefore, we adopted the optimized bilayer MoS_2_ models with a CO_2_ coverage of 3/4 ML to investigate the diffusion of metal ions.

**Fig. 2:**
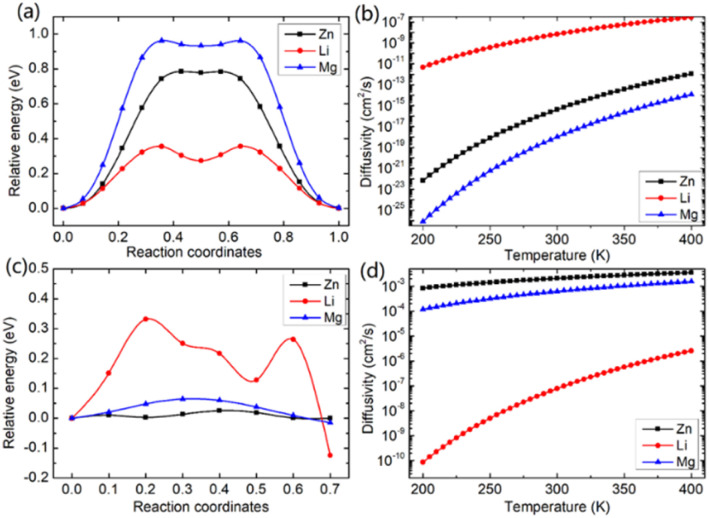
The computed diffusion barriers (a) and (c) and diffusivities (b) and (d) of metal Zn, Li, and Mg ions in pristine bilayer MoS_2_ and in bilayer MoS_2_ with an intercalating CO_2_ coverage of 3/4 ML, respectively.

In this study, we investigated the diffusion barriers of three metal ions (Zn, Mg, and Li) in bilayer MoS_2._ Based on the structure and 2H symmetry of the bilayer MoS_2_, we proposed two possible embedding sites (tetrahedral *T*_h_ and octahedral *O*_h_), as shown in Fig. S3† Considering the Zn ion as a representative, we calculated the relative energies of two possible embedding sites, and the results are shown in Fig. S4† and proposed the possible diffusion pathway of metal ions as (b) → (c) → (d), as shown in Fig. S4.† The diffusion barriers of Zn, Mg and Li ions in the pristine bilayer MoS_2_ were calculated, and the results are shown in Fig. 2a. The calculated diffusion barriers of Zn, Mg and Li ions in the pristine bilayer MoS_2_ are 0.785, 0.942 and 0.356 eV, respectively. The temperature-dependent ion diffusivity of the three metal ions was predicted based on the computed diffusion barriers considering thermal corrections (ESI†), and the results are shown in Fig. 2b. The calculated ion diffusivities are 4.18 × 10^−16^ cm^2^ s^−1^, 1.01 × 10^−16^ cm^2^ s^−1^ and 6.90 × 10^−9^ cm^2^ s^−1^ at room temperature, corresponding to the Zn, Mg, and Li ions, respectively. These calculation results indicate that the pristine bilayer MoS_2_ is a cathode material with limited performance for metal ion batteries. Clearly, new strategies are highly demanded to reduce the ion diffusion barrier for high-performance batteries.

We further demonstrated a feasible and effective strategy to reduce the ion diffusion barrier by using intercalating CO_2_ molecules to expand the MoS_2_ interlayer spacing. With a high coverage of 3/4 ML CO_2_, the interlayer spacing of MoS_2_ is expanded to 9.383 Å. Compared with the interlay spacing of 6.369 Å obtained from the pristine bilayer MoS_2_, a bigger interlayer spacing results in a weak interaction between layers and lower diffusion barriers of metal Li, Zn and Mg ions. With the intercalating CO_2_ molecules, the results shown in Fig. 2c and d reveal that the diffusion barriers of Zn and Mg were dramatically reduced to 0.026 and 0.064 eV, significantly boosting the Zn and Mg ion diffusion by 12 and 14 orders of magnitude at room temperature, while only by one order of magnitude for Li ions. Our comprehensive DFT results reveal that the CO_2_ intercalated MoS_2_ is a promising cathode material for realizing rapidly chargeable Zn and Mg metal ion batteries. Compared with previously reported results shown in Table 1, the calculated diffusivity of Zn in the bilayer MoS_2_ is much smaller than the experimental one in the bulk MoS_2_,^51^ and Mg has a higher diffusivity in the bilayer MoS_2_ than in the bulk MoS_2_. As for Li ions, they have nearly the same diffusivity in the bilayer MoS_2_ as in the bulk MoS_2_. It should be noted that the diffusivities of three ions are very low, showing that the pristine bilayer and bulk MoS_2_ are not suitable for metal ion batteries. Comparing the experimental values shown in Table 1 and our theoretical values reveal that the CO_2_ intercalated MoS_2_ has significant potential for metal ion batteries. However, Zn and Mg ions have much higher diffusivity in the CO_2_ intercalated MoS_2_ than in O-modified^51^ and MoS_2_/graphene heterojunctions.^52^ Not surprisingly, no significant improvement in Li ion diffusivity was found in our study compared with W and Mo alloyed MoS_2_.^53^

**Table 1:** The comparison between the computed ion diffusivity (cm^2^ s^−1^) with reported experimental values shown in brackets

Ions	Bilayer MoS_2_	Bulk MoS_2_	CO_2_–MoS_2_	Modified MoS_2_
Zn	4.18 × 10^−16^	(8 × 10^−12^)	2.08 × 10^−3^	(9 × 10^−8^ to 10^−9^)^51^
Mg	1.01 × 10^−18^	(1 × 10^−20^)	6.26 × 10^−4^	(3.24 × 10^−9^)^52^
Li	6.90 × 10^−9^	(3.78 × 10^−9^)	7.84 × 10^−8^	(1.2 × 10^−8^)^53^

**Fig. 3:**
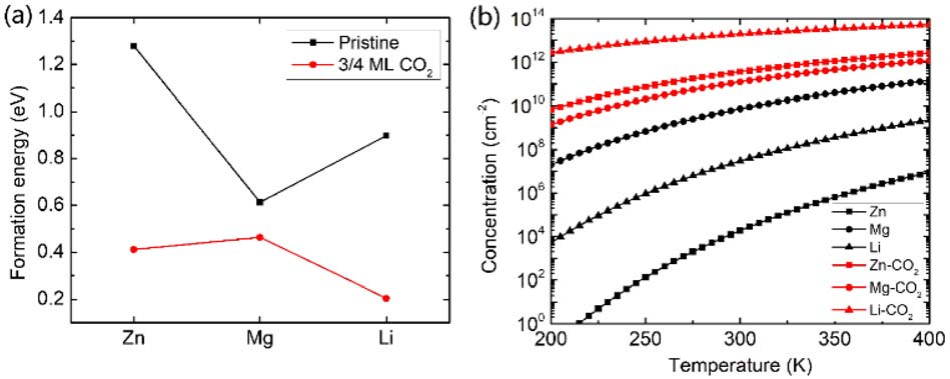
The intercalation energies (a) and temperature-dependent concentration (b) of metal ions of Zn, Mg and Li in the pristine and modulated bilayer MoS_2_ with an intercalating CO_2_ coverage of 3/4 ML.

Beyond the metal ion diffusion barrier and diffusivity, another challenge is to improve the storage capacity of AMMIBs, mainly determined by the embedding concentration of ions. Meanwhile the ion concentration or solubility in cathode materials is governed by the intercalation energy of metal ions.^49^ Consequently, we calculated the intercalation energy of embedded metal ions in the pristine and the intercalating CO_2_ bilayer MoS_2_ (ESI†). The results are shown in Fig. 3a reveal that the intercalation energy of Zn ion significantly decreases to 0.411 eV from 1.279 eV in the pristine bilayer MoS_2_, and the intercalation energy of Li dramatically decreases to 0.204 eV from 0.898 eV. However, the intercalation energy of Mg slightly decreases to 0.463 eV from 0.613 eV. Based on the calculated intercalation energies of metal ions in the pristine and CO_2_ embedded bilayer MoS_2_, we computed the temperature-dependent intercalation ion concentration (ESI†), and the results are shown in Fig. 3b. The Zn ion embedding concentration is enhanced by 7 orders of magnitude to 3.63 × 10^11^ cm^−2^, and the Li ion embedding concentration is enhanced by 5 orders of magnitude to 1.98 × 10^13^ cm^−2^ at 300 K. However, the Mg ion embedding concentration is slightly boosted by just one order of magnitude to 1.25 × 10^11^ cm^−2^. In contrast, the intercalating CO_2_ molecules have significant contributions to the enhancement of the embedding concentration of Zn and Li ions but have a feeble effect on Mg ions. Hence, we consider that the intercalation of CO_2_ molecules significantly promotes bilayer MoS_2_ to be a promising cathode material for high-capacity Zn and Li ion batteries, but only slightly for Mg ion batteries.

**Fig. 4:**
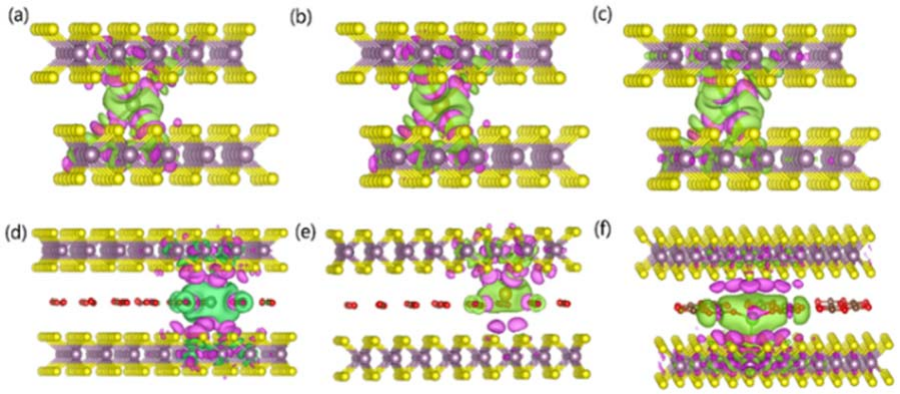
Charge density difference isosurfaces for (a) Zn, (b) Mg and (c) Li interacting with pristine MoS_2_ and the charge density difference isosurfaces for (d) Zn, (e) Mg and (f) Li interacting with CO_2_ embedded MoS_2_. The purple (green) color corresponds to charge accumulation (depletion). The isosurface is taken as 6 × 10^−5^*e*/Å.

**Fig. 5:**
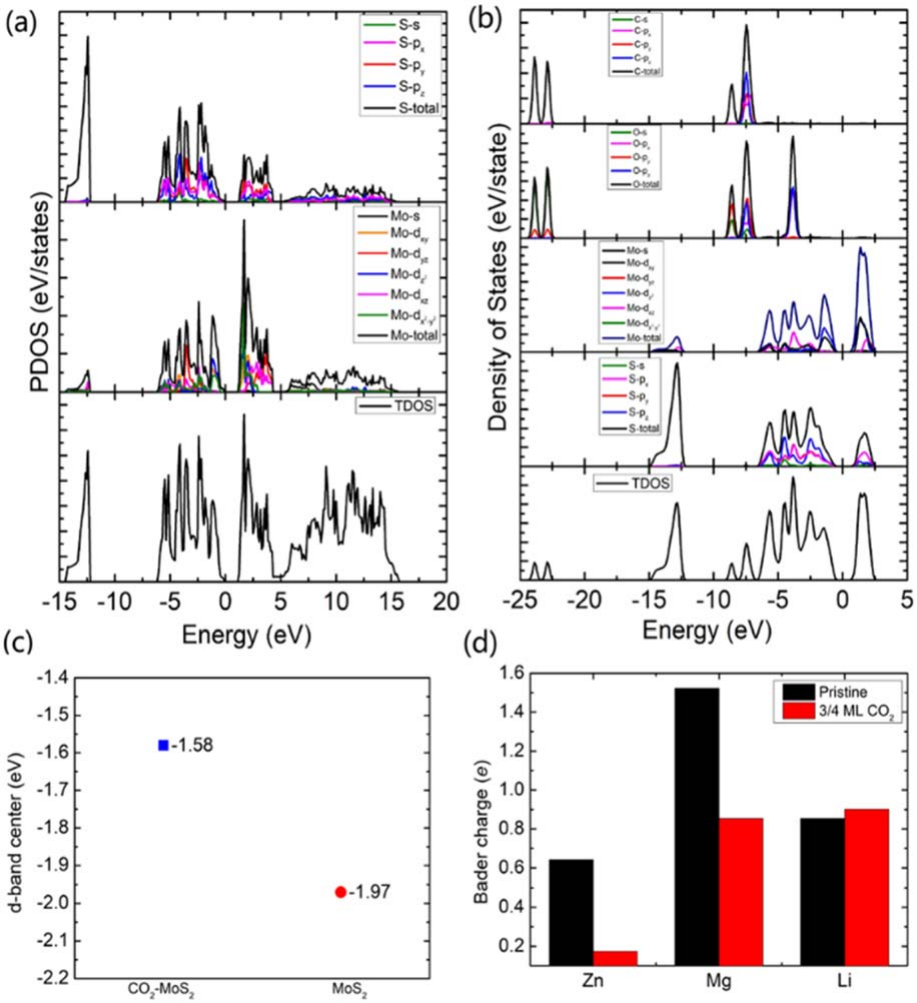
The calculated projected density of states (PDOS) of the pristine MoS_2_ (a) and CO_2_ intercalated MoS_2_ (b). (c) The calculated Mo d-band center with respective to the Fermi level (set to zero) of CO_2_ intercalated and pristine MoS_2_. (d) The calculated Bader charge of metal ions in the pristine and CO_2_ intercalated (3/4 ML) MoS_2_.

To gain an in-depth understanding of the diffusion barrier decrease originating from the intercalating CO_2_ and the possible chemical reaction between CO_2_ and embedding metal ions, we analyzed the charge difference between embedding metal ions and bilayer MoS_2_. As a benchmark, the charge differences of metal ions in the pristine bilayer MoS_2_ were evaluated as well. We calculate the charge density difference between the embedding ions and CO_2_ molecule intercalated bilayer MoS_2_ systems by using the formula of Δ*ρ* = *ρ*_(host+M)_ − (*ρ*_host_+*ρ*_M_), where *ρ*_(host+M)_, *ρ*_host_ and *ρ*_M_ represent the charge density of the ion embedded MoS_2_, the pristine MoS_2_ and the isolated metal ion, respectively. The simulation results are shown in Fig. 4, in which the purple color corresponds to the charge accumulation forming bonding states, and the green color represents the charge depletion forming anti-bonding states. Fewer bonding states and more anti-bonding states make the interaction stronger between embedded ions and their host systems. For the Zn ion embedding system as shown in Fig. 4a and d, the intercalating CO_2_ molecules result in more charge depletion than in the pristine MoS_2_, resulting in a decrease of the Zn ion intercalation energy. A similar tendency is also found in the Li ion embedding system shown in Fig. 4c and f. However, no significant change can be seen in the Mg ion embedding system shown in Fig. 4b and e. The results shown in Fig. 4 indicate that the intercalation of CO_2_ molecules has a great contribution to the intercalation energy of Zn and Li ions only, consistent with the calculated intercalation energies of metal ions as shown in Fig. 3a. Also, we found no electron transfer and bond formation between intercalating CO_2_ and embedded Zn and Mg ions, except for the Li-ion system. We also calculated the projected density of states (PDOS) of the pristine and intercalated CO_2_ (3/4 ML) MoS_2_ as shown in Fig. 5a and b, respectively. It can be seen that O-p orbitals have strong interaction with Mo-d orbitals. The results reveal that CO_2_ can be stabilized in the bilayer MoS_2_. We also computed d-band centers of Mo from the intercalated CO_2_ (3/4 ML) and pristine MoS_2_, and the results are shown in Fig. 5c. Intercalated CO_2_ molecules push the d-band of Mo to a higher energy state and result in a lower d-band center than in the pristine MoS_2_, and further enhance the metal ion solubility in intercalating CO_2_ bilayer MoS_2_. Bader charge^54^ provides the definition of the chemical bond for charge analysis, which is based on the electronic charge density. In Fig. 5d, the Bader charges for Zn and Mg are significantly decreased but there is a little increase for Li ions with CO_2_ embedding. The result reveal that intercalating CO_2_ molecules weaken the ion interaction of Zn and Mg with host elements, while the slightly increased Bader charge of Li ions reveals that Li ions have strong chemical interaction with CO_2_ as shown in Fig. 4f, which well explains the multi-saddle points in Li NEB results shown in Fig. 2c. We consider that those embedding CO_2_ molecules would not react with Zn and Mg and may form chemical bonds with Li ions. Therefore, we believe that it is feasible to use CO_2_ intercalation for improving the performance of MoS_2_-based Zn and Mg ion batteries.

To gain an in-depth understanding of the location of CO_2_ insertion at active sites, we computed the work function of pristine and CO_2_ with/without metal ion intercalation bilayer MoS_2_. The work function *Φ* is defined as the vacuum energy respective to the Fermi level (*Φ* = *E*_vac_ − *E*_f_). The work function of bilayer MoS_2_ highly depends on the crystal orientation, the presence of impurities, defects, and doping. The calculated local potentials along the *z*-direction are shown in Fig. S8a.† It should be noted that the results shown in Fig. S8a† are simply plotted in one figure without considering the common alignment reference. Based on the calculated local potential along the *z*-direction shown in Fig. S8a,† we computed the work functions of selected five cases and the results are shown in Fig. S8b.† It can be seen that the calculated work function of the pristine bilayer MoS_2_ is 6.02 eV, and the same as that of 3/4 ML CO_2_ embedded MoS_2_. The results reveal that intercalated CO_2_ molecules do not affect the work function. Therefore, we considered that CO_2_ is a promising material to expand the interlay spacing of layered MoS_2_. With metallic ion (Zn, Mg and Li) insertion, the work function decreases to 5.65 eV in Zn intercalated 3/4 ML CO_2_ embedded MoS_2_, further decreases to 4.78 eV in Mg intercalated 3/4 ML CO_2_ embedded MoS_2_, and further decreases to 4.53 eV in Li intercalated 3/4 ML CO_2_ embedded MoS_2_.

## Conclusions

In conclusion, we developed a simple and effective strategy by tuning the interlayer spacing of MoS_2_ and the intercalation energy of Zn, Mg and Li ions for cathode materials of rapidly chargeable batteries. Employing DFT simulations, we demonstrate that the intercalation of CO_2_ molecules with a 3/4 ML coverage effectively expands the interlayer spacing, reduces layer coupling of bilayer MoS_2_, and significantly reduces the diffusion barrier and intercalation energies of metal ions, achieving high performance metal ion batteries. As a result, the diffusivities of Zn and Li increase by 12 and 13 orders of magnitude, and the intercalating ion concentration or the storage capacities of Zn and Li ion batteries are boosted by 7 and 5 orders of magnitude. Our simulation result demonstrated that intercalated CO_2_ molecules significantly contribute to both diffusivity and embedding concentration of Zn ions but they contribute to only the diffusivity of Mg and the embedding concentration of Li ions. Therefore, we demonstrate that CO_2_ molecule intercalated bilayer MoS_2_ is a promising cathode material for high-capacity and rapid-charging ZIBs. The strategy of expanding interlay spacing and reducing intercalation energy developed in this study can be generally applied to increase the ion storage capability in layered structure-based electrode materials and sheds light on the development of advanced materials for next-generation high-performance energy storage.

## Author contributions

Xuefei Han: methodology, investigation, writing – original draft, and writing – review & editing. Jing Yang: writing – review & editing. Yong-Wei Zhang: conceptualization and writing – review & editing. Zhi Gen Yu: conceptualization, methodology, writing – review & editing, and supervision.

## Conflicts of interest

There are no conflicts to declare.

## Supplementary Material

NA-005-D3NA00068K-s001

NA-005-D3NA00068K-s002

NA-005-D3NA00068K-s003

NA-005-D3NA00068K-s004

NA-005-D3NA00068K-s005

NA-005-D3NA00068K-s006

NA-005-D3NA00068K-s007

NA-005-D3NA00068K-s008

NA-005-D3NA00068K-s009

NA-005-D3NA00068K-s010

NA-005-D3NA00068K-s011

NA-005-D3NA00068K-s012
