# Supplementary material for: Molecular engineering on a MoS2 interlayer for high-capacity and rapid-charging aqueous ion batteries
Source: Nanoscale Adv. 2023 Apr 4;5(9):2639–45. doi: 10.1039/d3na00068k (PMC10153098; doi:10.1039/d3na00068k)
Supplement: NA-005-D3NA00068K-s006 [file NA-005-D3NA00068K-s006.pdf]

Electronic Supplementary Material (ESI) for Nanoscale Advances.
This journal is © The Royal Society of Chemistry 2023

Supplementary materials

Molecular Engineering on MoS_2 Interlayer for High-Capacity and Rapid-Charging Aqueous Ion Batteries

XBauefei Han,[a,b,c] Jing Yang,[a] Yong-Wei Zhang[a,b] and Zhi Gen Yu*[a,b]

This supporting file includes:

Computational Methods; Metal ion diffusivity and intercalation concentration calculations

Figs. S1 to S7; References 1-6

Movies S1 to S10 (S1 (Zn-pristine_MoS_2_neb.mp4), S2 (Mg-pristine_MoS_2_neb.mp4), S3(Li-pristine_MoS_2_neb.mp4), S4 (Zn-CO_2_MoS_2_neb.mp4), S5 (Mg -CO_2_MoS_2_neb.mp4), S6 (Li-CO_2_MoS_2_neb.mp4), S7 (CO_2_armchair.mp4), S8 (CO_2_zizgag.mp4), S9 (CO_2_crossing.mp4)), S10 (1CO_2.mp4), S11(1ML.mp4).

[a]Institute of High Performance Computing (IHPC), Agency for Science, Technology and Research (A*STAR), 1 Fusionopolis Way, #16-16 Connexis, Singapore 138632, Republic of Singapore.
[b]Department of Materials Science and Engineering, National University of Singapore, Singapore 117575, Singapore.
[c]AVIC Xi'an Flight Automatic Control Research Institute, 710065, China.

1. Computational method

All calculations were carried out using the density functional theory (DFT) with the generalized Perdew-Burke-Ernzerhof (PBE)[1] and the projector augmented-wave (PAW) pseudopotential plane-wave method[1] as implemented in the VASP code.[3] A 10×10×1 Monkhorst-Pack (MP) *k*-point grid was used for bilayer *AA*′ stacking MoS_2 unitcell optimization calculations and a plane-wave basis set with an energy cutoff of 500 eV. Good convergence was obtained with these parameters and the total energy was converged to 1.0×10^{-6} eV per atom, as well as the stress exerted on the cell was less than 0.1 kbar and the forces exerted on the atoms were less than 0.01 eV/Å. In this study, we carried out calculations with the van der Waals (vdW) correction by employing optPBE-vdW functional[4] using a 2×2×1 MP *k*-point grid and 5×5×1 supercells with a vacuum separation of 20 Å containing 50 Mo and 100 S atoms. We employed optPBE-vdW functional to consider the van der Waals force between layers, which is described in Computational method (Supporting information). We adopted optPBE-vdW functional based on the reported results, in which CO_2 adsorption energies obtained using revPBE and optPBE correlations are similar.[5]

2. Diffusivity calculation

Based on the calculated diffusion barriers, we calculated the metal ions diffusivity by using the transitional state theory with the Arrhenius relationship[6]

$$D = D^0 \exp\left(- \frac{\Delta H}{K_B T} \right), \quad (1)$$

where D^0 is the pre-exponential factor of diffusion, ΔH is the activation enthalpy of diffusion, K_B is the Boltzmann constant and T is the absolute temperature. Considering all the vibrational frequencies of the diffusion ion, the ion diffusivity can be calculated by:

$$D = d^2 \frac{K_B T}{h} \exp\left(- \frac{\Delta G}{K_B T} \right), \quad (2)$$

where, d is the distance between the initial state and the final state of diffusion ion, h is the Planck's constant, and ΔG is the Gibbs free energy of diffusion, which can be calculated by using $\Delta G = \Delta_H + \Delta E_{zpe} - T\Delta S$, where ΔE_{zpe} and ΔS are the change of the zero-point energy and the entropy, respectively. In our study, we just considered the vibration contribution to the entropy without considering the change of system volume ($\Delta V = 0$), the Gibbs free energy of diffusion can be specialized as $\Delta G = \Delta_H + \Delta F_{vib}$, where, F_{vib} denotes the vibrational Helmholtz free energy, which can be calculated by $F_{vib} = \sum_i \left[\frac{1}{2}\hbar\omega_i + K_b T.\ln\left(1 - exp\left(- \frac{\hbar\omega_i}{K_b T} \right) \right) \right]$. Here, $\hbar$ is the reduced Planck's constant and ω_i is the *i*-th vibrational frequency of the diffusing ion. The initial and transition states of nudged elastic band (NEB) calculations were considered to calculate the vibration frequencies for the free energy corrections.

3. Ion intercalation energy calculation

The intercalation energy of an embedded ion E_f can be calculated based on the equation of $E_f = E_{(Host + M)} - (E_{Host} + \mu_M)$. Here, $E_{(Host + M)}$, E_{Host} and E_M are the energy of metal ions embedded bilayer MoS_2, ion-free bilayer MoS_2 and the chemical potentials of metal ions obtained from the bulk unitcell of metallic Zn, Mg and Li. The metallic Zn and Mg unitcells with the hexagonal close packed crystal structure and the metallic Li unitcell with body-centered cubic (bcc) structure were optimized to obtain the chemical potentials of metal elements. The intercalation ion concentration can be calculated based on $N = N_0 * exp(\frac{-E_f}{K_b T})$, where N_0 is the maximum ion embedding site concentration, E_f is the intercalation energy directly from DFT calculation, K_b is the Boltzmann constant and T is the temperature.

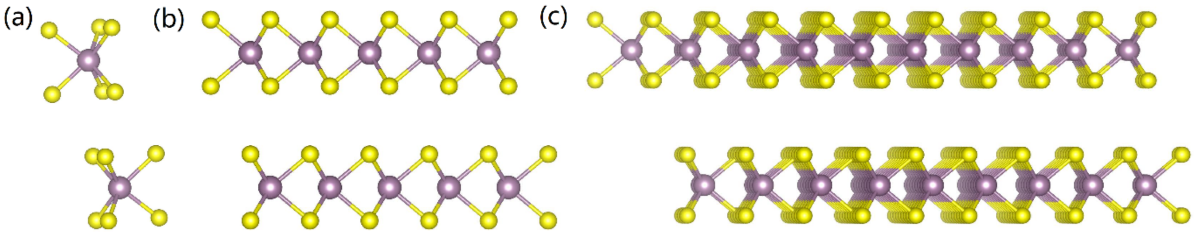

Figure S1. Atomic models of bilayer MoS_2. (a) *AA′* stacking unitcell. (b) 5×5×1 supercell. (c) Rectangle supercell. The yellow and light blue balls represent S and Mo atoms, respectively.

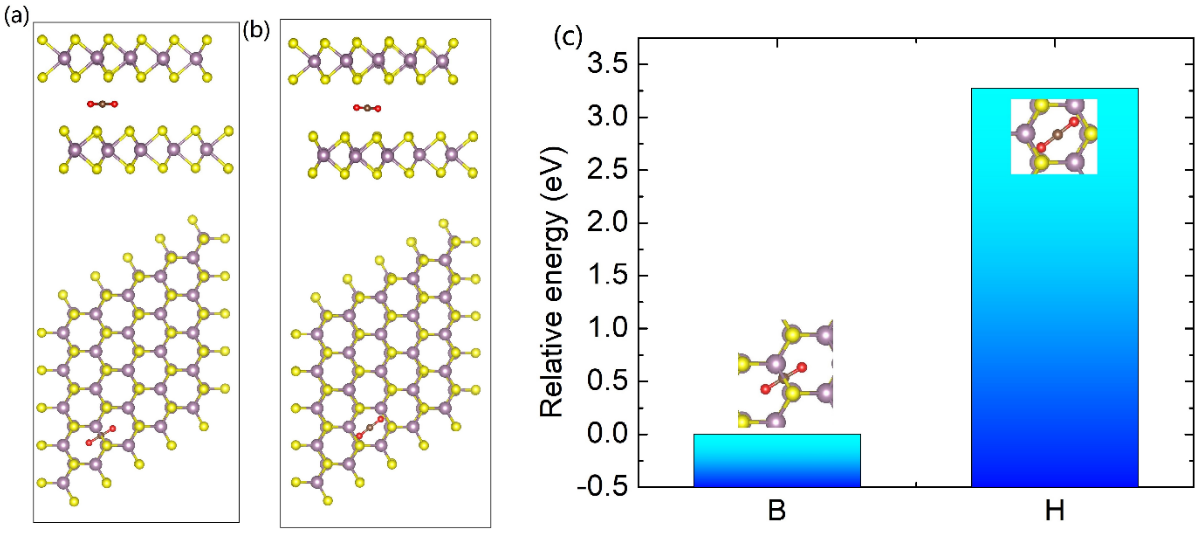

Figure S2. The optimized models of one intercalating CO_2 molecule in bilayer MoS_2. (a) Bridge site. (b) Hollow site. (c) The calculated relative energies of two possible embedding sites.

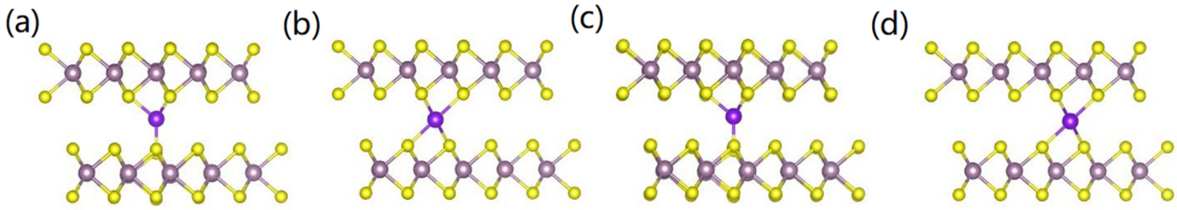

Figure S3. Two possible embedding sites for metal ions. (a) and (c) Tetrahedral T_h site. (b) and (d) Octahedral O_h site. The purple ball represents the metal ion. The possible diffusion pathway of (b)→(c)→(d) was suggested.

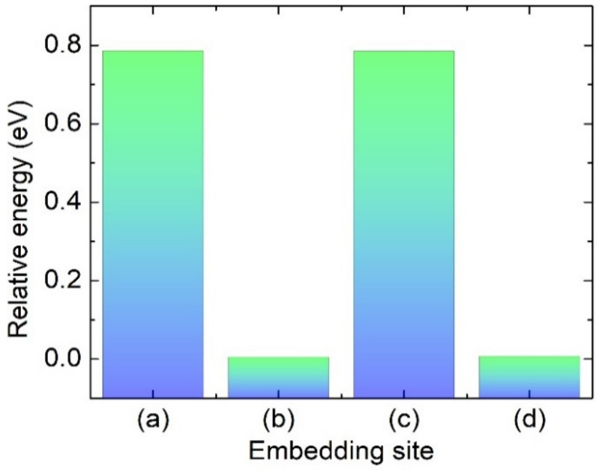

Figure S4. The calculated relative energies of two possible embedding sites and two symmetry sites.

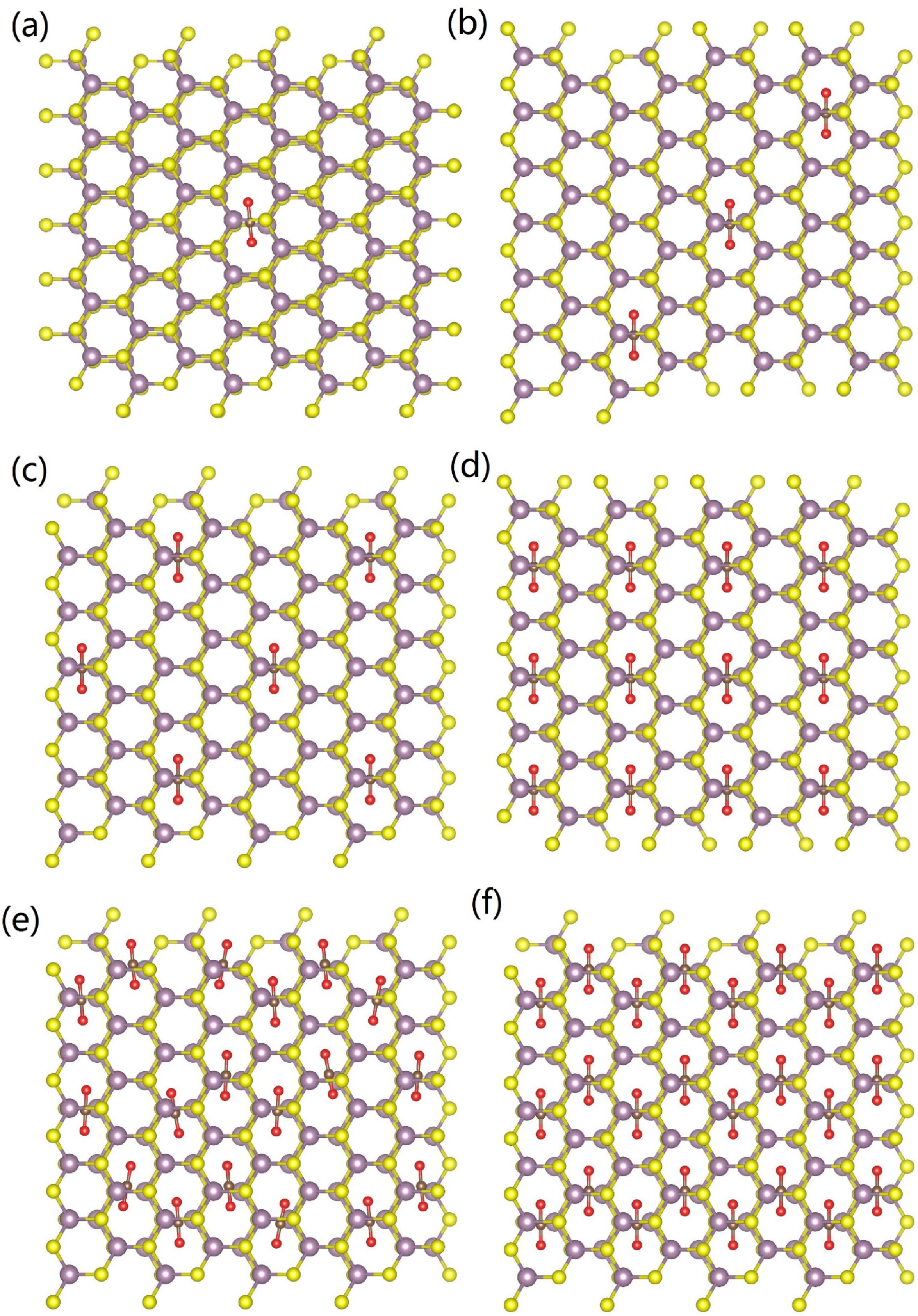

Figure S5. The optimized different coverages of the intercalating CO_2 in bilayer MoS_2. (a) One intercalating CO_2 molecule. (b) 1/8 ML. (c) 1/4 ML. (d) 1/2 ML. (e) 3/4 ML. (f) 1ML.

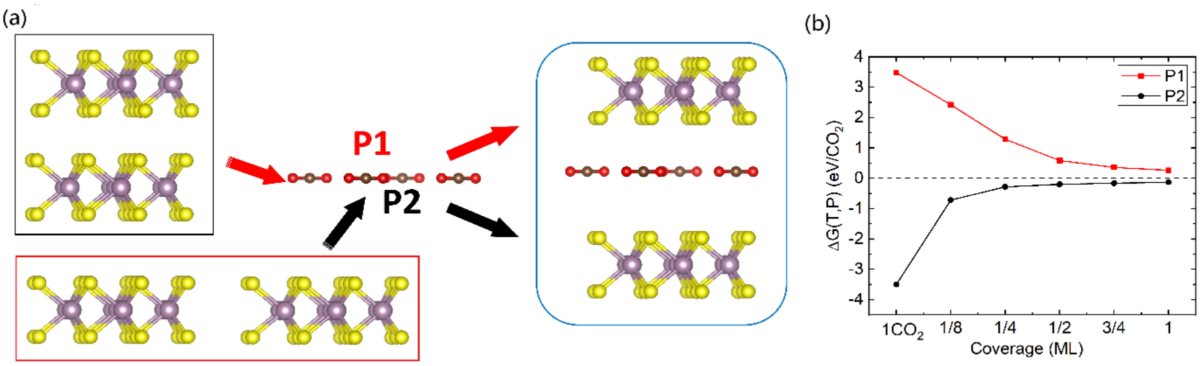

Figure S6. (a) Two proposal pathways for the fabrication of CO_2 intercalated MoS_2. (b) The calculated stacking energy of intercalating CO_2 in bilayer MoS_2 with two possible pathways intercalating CO_2 into MoS_2.

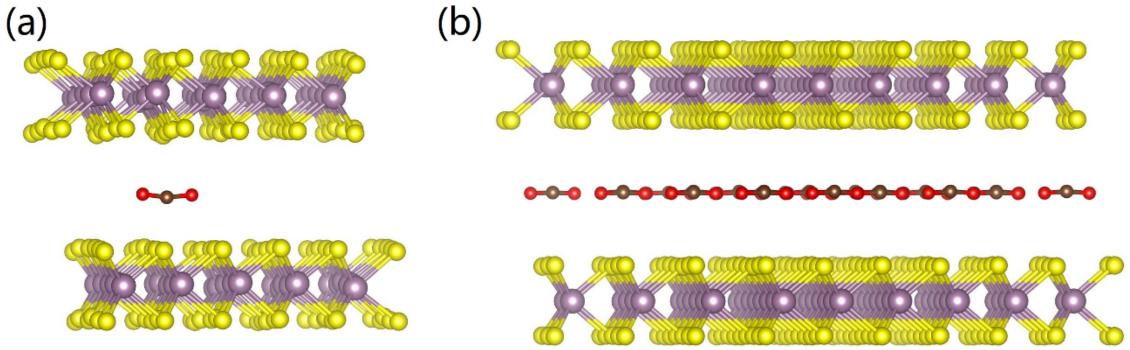

Figure S7. The optimized atomic models with a vacuum thickness of 10 Å from AIMD simulations. (a) One CO_2 molecule intercalated bilayer MoS_2. (b) Bilayer MoS_2 with 1ML coverage of CO_2.

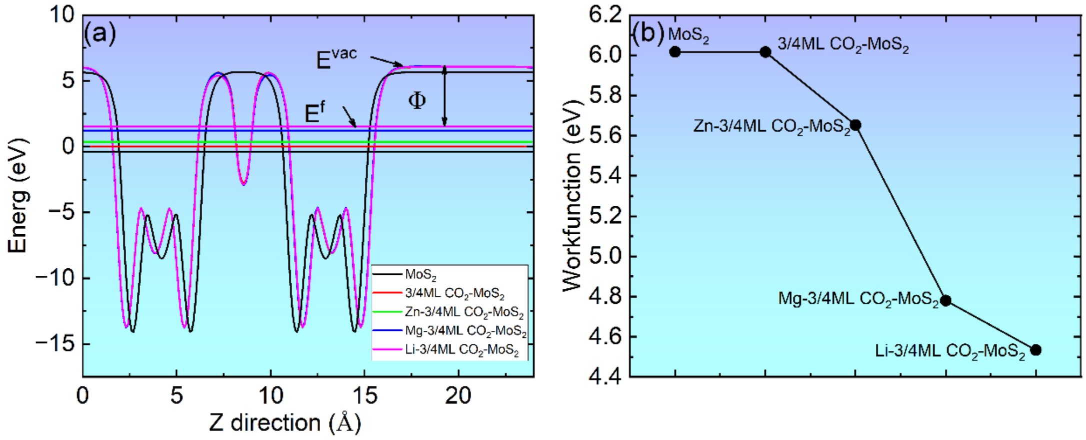

Figure S8. (a) The calculated local potentials (a) and work functions (b) of MoS_2, ¾ ML CO_2 embedded MoS_2 and metals (Zn, Mg and Li) intercalating ¾ ML CO_2 embedded MoS_2. One CO_2 molecule intercalated bilayer MoS_2.

References

(1) J. Perdew, K. Burke and M. Ernzerhof, *Phys. Rev. Lett*., 1996, ***77***, 3865.

(2) P. Blöchl, *Phys. Rev. B: Condens. Matter Mater.* Phys., 1994, **50**, 17953.

(3) G. Kresse and J. Furthmüller, *Comput. Mater. Sci.,* 1996, ***6***, 15–50.

(4) J. Klimeš, D. R. Bowler and Angelos Michaelides, *Phys. Rev. B: Condens. Matter Mater. Phys.,* 2011, ***83***, 195131.

(5) F. M. Enujekwu, C. I. Ezeh, M.W. George, M. Xu, H. Do, Y. Zhang, H. Zhao and T. A. Wu, *Nanoscale Adv.,* 2019, *1*, 1442-1451.

(6) H. Eyring, *J. Chem. Phys*., 1935, ***3***, 107–115.
